# Reversible cerebral vasoconstriction syndrome: literature review

**DOI:** 10.1186/s41983-023-00607-9

**Published:** 2023-01-11

**Authors:** Michelle Zonkowski Ribas, Gabriela Ferreira Paticcié, Sara Diógenes Peixoto de Medeiros, Arthur de Oliveira Veras, Felipe Micelli Noleto, Júlio César Claudino dos Santos

**Affiliations:** 1 Federal University of Paraná (UFPR), Curitiba, Paraná Brazil; 2 Faculty of Medicine, Federal University of Juiz de Fora, Juiz de Fora, MG Brazil; 3 Facisa University Center, UNIFACISA, Campina Grande, PB Brazil; 4 Clinical Hospital of the Faculty of Medicine of Ribeirão Preto - USP, Ribeirão Prêto, SP Brazil; 5 Faculty of Medicine, Christus University Center, UNICHRISTUS, Fortaleza, CE Brazil; 6 Neurosciences Laboratory, Department of Neurology and Neurosurgery, Federal University of São Paulo, Sena Madureira, 1500, Vila Clementino (SP), São Paulo, SP 04021-001 Brazil

**Keywords:** Reversible cerebral vasoconstriction syndrome, Thunderclap headache, Cerebral vasculitis

## Abstract

Reversible Cerebral Vasoconstriction Syndrome (RCVS) is a neurovascular condition characterized by a severe sudden-onset headache that may be associated with focal neurological deficits. On imaging, the suggestive finding corresponds to multifocal vasoconstriction of the cerebral arteries, with a spontaneous resolution of approximately 12 weeks. The identification of precipitating factors and diagnosis must be carried out early, so that adequate management is established and the patient has a good prognosis, given the risk of secondary complications and residual neurological deficits. This study consists of a literature review based on the analysis of articles published between 2017 and 2022 in PubMed, SciELO, and ScienceDirect on RCVS, intending to understand the clinical and radiological characteristics, diagnosis, treatment, and prognosis of patients with RCVS. The pathophysiology, drug management, and prognosis still lack solid evidence; therefore, further studies on RCVS are needed to expand medical knowledge and avoid underdiagnosis and inadequate treatment of this important condition.

## Introduction

The Reversible Cerebral Vasoconstriction Syndrome (RCVS) is characterized by sudden headache and reversible multifocal cerebral vasoconstriction [1–4]. It is more prevalent in women [5–8], mainly because it often has puerperium as a precipitating factor [7, 9–11].

The most common manifestation is thunderclap headache, which has a sudden onset and peak intensity in 1 min [5, 9, 12, 13], and may be accompanied by nausea, vomiting, photophobia, and phonophobia [5, 8, 10]. In addition, focal neurological deficits may occur in 8–43% [1, 14] and epileptic seizures in up to 17% of patients with RCVS [1].

Reversible Cerebral Vasoconstriction Syndrome should be suspected when there is a history of sudden headaches with normal physical examination, cranial tomography (CT), and magnetic resonance imaging (MRI) of the head [9, 12]. Given this situation, angiography should ideally be performed to detect multifocal cerebral arterial vasoconstriction [1, 15–17]. It is necessary to demonstrate the reversibility of cerebral vasoconstriction within 12 weeks to confirm the diagnosis [16–18].

Once the diagnosis is established, treatment should be initiated, mainly by discontinuing the triggering factor, if known [9, 13, 19], and using medications, such as calcium channel blockers (CCBs) [5, 20], avoiding complications, which may be present in up to a third of patients [3, 13, 14], as well as residual neurological deficits [17].

As the wrong or delayed diagnosis of RCVS usually results in unnecessary diagnostic tests, deleterious treatments, and increases the risk of neurological sequelae [3, 5, 21], to reduce mortality, early diagnosis and treatment are essential [13, 22, 23]. Thus, a greater understanding of the disease is necessary, in which we present an extensive literature review on this topic.

## Methods

This article presents a literature review based on scientific articles published from 2017 to 2022 in PubMed, SciELO, and ScienceDirect on RCVS.

**Fig. 1:**
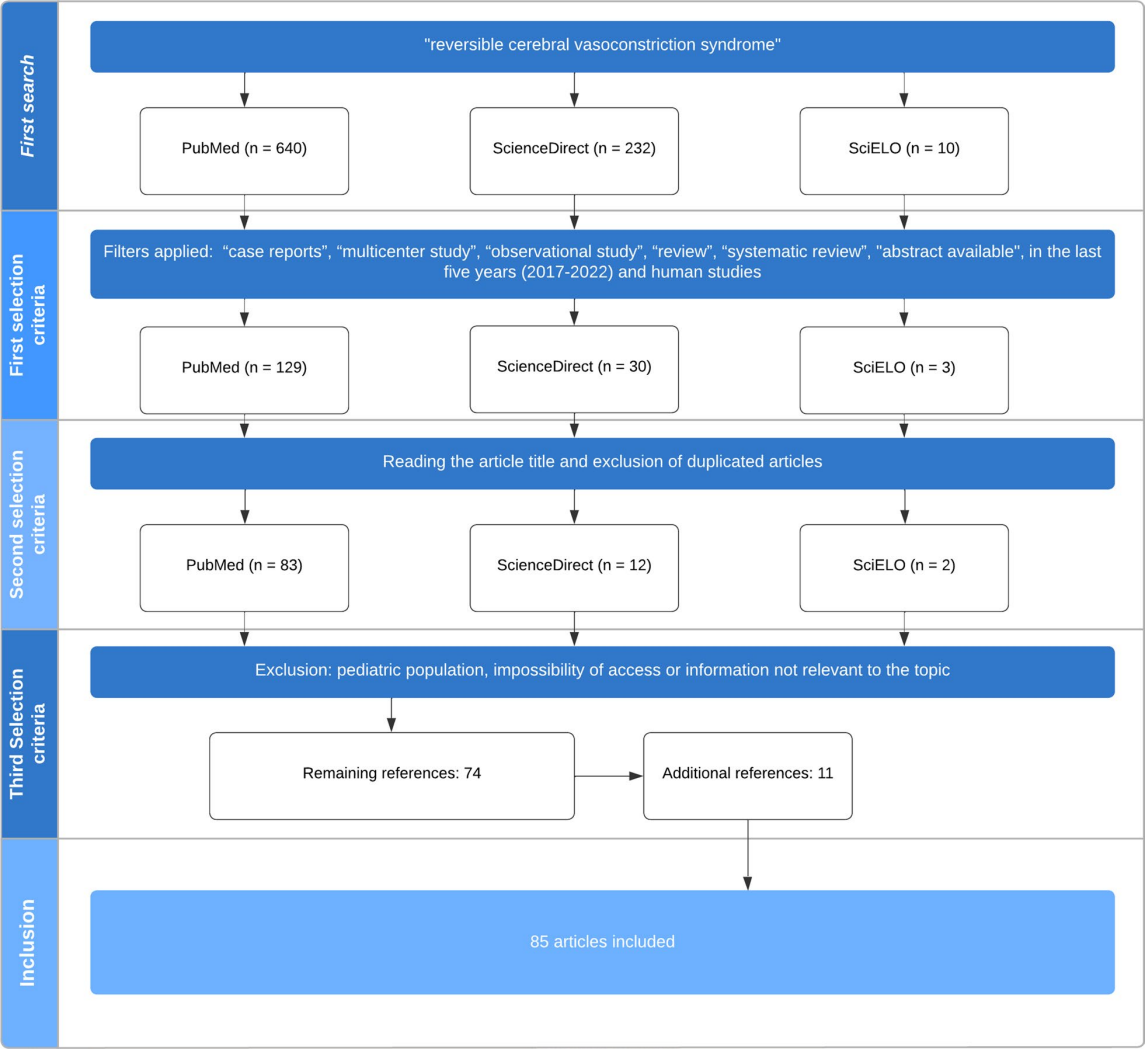
Article selection flowchart

In the first step of the methodology, a search was carried out in Pubmed, SciELO, and ScienceDirect databases with the title “reversible cerebral vasoconstriction syndrome” and the filters “case reports”, “multicenter study”, “observational study”, “review”, and “systematic review” were applied. In addition to these filters, only studies involving humans and those published in the last 5 years were selected. The article should be available and in English. A total of 129 articles were found in PubMed, three in SciELO, and 30 in ScienceDirect (Fig. 1).

In the second stage, after reading the title and excluding duplicate articles, 83 articles were selected from PubMed, two from SciELO, and 13 from ScienceDirect.

The third stage of selection involved the exclusion of articles involving only the pediatric population (12 articles), those with an impossibility of access (seven articles), or those that did not contain information relevant to the topic (four articles). A total of 74 articles remained at the end of this stage.

In addition, 11 new articles were selected and manually added according to their relevance to the study. Therefore, a total of 85 articles were included in this study.

## Discussion

### General aspects

Reversible Cerebral Vasoconstriction Syndrome, also known as Call-Fleming Syndrome, postpartum angiopathy, migrainous vasospasm, and benign central nervous system angiopathy [10, 24, 25], is a syndrome characterized by thunderclap headache with severe and transient vasoconstriction of medium-sized vessels of the circle of Willis or extracranial circulation [1, 3, 12, 26]. It commonly manifests with severe headache with or without the presence of ischemia or other acute neurological symptoms [1, 2, 7, 12, 13, 19–21, 27–32].

Radiologically, the disease is characterized by multifocal vasoconstriction of the cerebral arteries that can lead to multiple infarctions and usually resolves spontaneously within 3 months [6, 8, 12, 13, 17, 19, 26–28, 30, 33–37]. It can affect any age group, including children [10] and young adolescents [7]. However, it primarily affects individuals between 20 and 50 years of age [1, 2, 9–11, 13, 20, 38, 39], with a peak around 42 years of age [7, 40]. It is more common in women than in men, with incidence rates of 2.6:1 to 10:1 [1, 5–7, 10, 12, 17, 20, 28, 41–43]. In men, it usually manifests a decade earlier than the average described for women [10].

### Associated factors

The largest studies suggest that 50–80% of patients will have a secondary cause for RCVS [1, 10, 19, 35, 41, 44]. The main precipitating factors described are: postpartum state [7, 10, 13, 14, 16, 25, 31, 35, 39, 45–47], drugs with vasoactive properties [10, 12–14, 36, 39, 47, 48], such as cocaine, marijuana, and heroin [33, 39], catecholamine-secreting tumors [35, 49–51], autoimmune disorders, such as systemic vasculitis, systemic lupus erythematous, antiphospholipid syndrome [13], blood transfusions, ginseng [5, 6], sexual intercourse, temperature differences (baths too hot or too cold), air travel [39, 48], Corona Virus Disease 2019 (COVID-19) infection [36, 52–54] and medications [6, 29, 39, 48].

Nasal decongestants, immunosuppressants [9, 29, 48], antidepressants [39], oral contraceptive pills [6], indomethacin, and ergots, such as ergotamine tartrate, lisuride, methylergometrine and bromocriptine are some of the medications described in association with RCVS [6].

The puerperium is responsible for 10–50% of cases [7, 9–11, 45]. In one-third of cases, it is associated with another known factor, such as vasoconstrictors used in epidural anesthesia and postpartum hemorrhage [10, 55]. The history of migraine also proves to be a relevant risk factor, with a prevalence reported in the literature of 9.8–42% [10, 20, 56], an important finding, since the history of migraine increases the risk of bleeding complications in RCVS [9]. In addition, there are descriptions of the development of RCVS due to the use of medications utilized in the treatment of migraine, demonstrating the need to carefully choose the medications to be used in a migraine patient who already presented with RCVS [9].

Posterior reversible encephalopathy syndrome (PRES) is a disease that can occur simultaneously with RCVS and many studies suggest overlapping pathophysiological mechanisms [33, 41]. Furthermore, some authors have considered the possibility that PRES and RCVS are a continuum of reversible disorders of the cerebral vascular function [33].

### Pathophysiology

Although the cause of RCVS is not yet fully detailed in the literature, most studies point to a transient dysregulation in the control of brain vessel tone, which results in multifocal vasoconstriction [46, 57]. It is known that the control of vascular tone can be affected by several biochemical and immunological factors [2, 58–60], among which, in scientific evidence, endothelial dysfunction and sympathetic hyperstimulation stand out [3, 10, 13, 19, 20, 39, 61, 62] and alteration of vascular smooth muscle by oxidative stress [13, 19, 20, 39, 61, 63]. In addition, genetic predispositions such as Brain-Derived Neurotrophic Factor gene polymorphism [58] are also described and seem to be associated with more severe clinical conditions [13].

### Clinical presentation

Thunderclap headache has a reported prevalence between 95% and 100% in individuals diagnosed with RCVS [9, 10, 20] and may be the only clinical manifestation in 76–85% of patients [1, 10]. Characteristically, headache is severe, sudden onset, and peaks in approximately 1 min [5, 7, 9, 10, 12, 13, 46, 58]. In general, the pain presentation is bilateral and diffuse; however, in some cases, it can be localized [9, 10, 64]. In up to 94–100% of patients [1], headache episodes may recur within 1–3 weeks [5, 9, 12], with an average of four new attacks in the following 4 weeks [7]. In addition, headaches may be triggered or exacerbated by Valsalva maneuvers, such as coughing and sexual activity [1, 7, 9]. Less severe headaches of a constant nature may be present between episodes of thunderclap headaches [41].

In addition to headaches, patients often experience nausea, vomiting, photophobia, and phonophobia [5, 8, 10]. Focal neurological deficits may be present, with an estimated prevalence between 8% and 43% of cases [1, 14, 46] and include encephalopathy, visual changes, dysarthria, aphasia, ataxia, epileptic seizures, and focal numbness or weakness [5, 12].

Less frequently, RCVS may present with non-thunderclap and nonspecific headaches. It can be a single, recurrent, or progressive episode, and pain intensity varies from moderate to severe. There are also reports of patients who do not have any headaches [10, 65]. These atypical patients usually present with severe clinical symptoms and progress to stroke, severe posterior reversible encephalopathy syndrome, dizziness, mental confusion, or coma [10].

### Diagnosis

Reversible Cerebral Vasoconstriction Syndrome diagnosis is based on compatible clinical history, physical examination, and neuroimaging tests showing multifocal segmental vasoconstriction of cerebral arteries. Within 3 months, reversibility of this vasoconstriction occurs, evidenced in neuroimaging, however, some patients may persist with clinical deficits as well as complications, such as ischemic lesions and cerebral hemorrhage [9, 16, 17, 48, 62, 64].

The physical examination may be normal, but in some cases, there are neurological deficits that contribute to the suspicion, such as aphasia, hemiparesis, ataxia, and visual changes [20].

Complementary laboratory tests such as a blood count, electrolyte analysis, and liver function tests are usually normal. However, inflammatory markers may occasionally be elevated when associated with a precipitating disease. Therefore, serum and urine drug toxicology should also be performed to screen for substances precipitating vasoconstriction [64]. Cerebrospinal fluid (CSF) is usually normal. However, it may present some abnormalities, such as pleocytosis of up to 15 nucleated cells, a small number of red blood cells, and minimally increased protein level (< 100) [1, 33, 64].

As for imaging, digital subtraction angiography is the gold standard method, but it is invasive and can lead to complications [1, 9, 10, 13, 41], and its performance during pregnancy is contraindicated [16]. Resonance angiography and CT angiography are alternatives used [1, 14], despite the possibility of not detecting abnormalities in small-caliber vessels [9]. Studies suggest that arteries with smaller calibers are affected first [13, 20, 35, 41, 66], so imaging tests may be normal initially [3, 10, 13, 34, 41, 67]; therefore, early angiographic evaluation may be negative in up to 20% of patients with RCVS [3, 5, 34]. For this reason, it is recommended that angiography be performed within 2–3 weeks of the onset of symptoms, a period in which cerebral vasoconstriction is at its maximum [36, 67]. If there is a high clinical suspicion of RCVS and images without alterations, the diagnostic hypothesis cannot be ruled out [9, 68]; therefore, neuroimaging should be repeated in 1–2 weeks [41].

The characteristic diagnostic finding is the presence of “beading” or “pearls on a sting” in the cerebral arteries, which corresponds to the alternating pattern of intense vasoconstriction and dilatation observed [5, 9, 10, 14, 17, 20]. Another frequent finding in angiography is multifocal strictures [17, 69]. On CT and MRI, the most frequent findings are ischemic lesions and cerebral hemorrhage, especially subarachnoid hemorrhage (SAH) [17, 70]. Magnetic resonance imaging is beneficial in analyzing associations and complications of RCVS, such as stroke [10, 17].

Although there is still no universal diagnostic criterion established [16, 17, 62], some studies describe the use of scores for the diagnosis of RCVS. For example, the RCVS2 score uses clinical and imaging information to make a differential diagnosis with other arteriopathies [16]. Scores above five have a specificity of 99% and a sensitivity of 90% [71], and if the score is equal to nine, the diagnosis of RCVS can be defined [16]. In addition, other scoring systems have been developed and must be validated in the patient's country of origin to be used [20, 72]. An example is the RCVS–TCH, which was presented, in an initial study, with a sensitivity of 77% and specificity of 78% [20, 67].

### Differential diagnosis

Reversible Cerebral Vasoconstriction Syndrome is contained in the group of diseases known to have a thunderclap headache. This group includes stroke, SAH, cervical artery dissection, intracranial aneurysm, cerebral venous thrombosis, and hypertensive crisis [5, 20, 56, 73]. The differential diagnosis should also include Primary Angiitis of the Central Nervous System (PACNS), as the radiographic findings seen in RCVS are also present in PACNS, and there may be diagnostic confusion. Primary Angiitis of the Central Nervous System headache evolves insidiously and progressively, and the CSF often presents elevated protein, pleocytosis and, occasionally, oligoclonal bands [1, 5]. In RCVS, however, the headache is sudden, and the CSF is usually normal [1, 12]. At the time of clinical investigation, it is essential to make this differentiation carefully, since the recommended treatment of glucocorticoids used in PACNS can be harmful in RCVS, with worsening symptoms [1, 5, 48].

Posterior reversible encephalopathy syndrome is also part of the set of diseases present in the differential diagnosis of RCVS [9, 56, 68, 71]. Similar to RCVS, PRES may present with headaches [23]. Neuroimaging is an important exam to make this differentiation, as in PRES, vasogenic edema is seen in the parietal, frontal, and occipital regions [9, 68]. Another difference is that PRES rarely accompanies a stroke, as it happens with RCVS [71].

In addition, vasospasm secondary to SAH is also a differential diagnosis. Both RCVS and vasospasm secondary to SAH present severe headaches and occur more frequently in middle-aged females. On CT angiography, both conditions demonstrate subtle differences in segmental vasospasm [46].

### Treatment

The treatment needs, initially, to go through discontinuation of the triggering factor of the symptoms, if known, such as illicit drugs, aggressive vasoactive drugs, and Valsalva maneuvers [9, 12, 13, 46].

In drug treatment, according to case reports and expert analyses, symptomatic patients are recommended to use multimodal analgesia, CCBs, antiepileptics in case of epileptic seizures, and antiemetics as necessary [5, 20]. Non-steroidal anti-inflammatory drugs, in turn, are related to worsening RCVS symptoms and are, therefore, not recommended [20].

Regarding CCBs, intravenous and oral nimodipine, with a dosage of 30–60 mg every 4 h [64], is best described in the improvement of headache [74] in 64–83% of patients [1, 11, 17, 75]. In the form of an infusion of this drug, the dosage of one to two mg/h has been described. All routes of administration have been reported for the same 4–8 week period [64]. The use of nimodipine, at a dosage of 60 mg orally every 4 h, to avoid encephalopathy, intracranial bleeding, and reflex hypertension caused by cerebral vasoconstriction has also been described. It appears to be safe in breastfeeding women and neonates [22]. Moreover, in the context of COVID-19 infection, RCVS responds positively to nimodipine and aspirin [54].

However, although nimodipine is usually the first choice for the treatment of RCVS, it has not been shown to improve long-term outcomes or prevent complications [5, 9, 13, 20, 41].

The other choices of CCBs are verapamil and nicardipine [11, 76] orally and for 4–8 weeks [46]. In the literature, intra-arterial (IA) verapamil has been reported in a patient with RCVS refractory to treatment, causing a profound reversal of vasospasm. On the other hand, nimodipine was administered only to prevent further vasospasm. In this approach, the patient was discharged in a stable condition when it was verified that his weakness improved significantly [46]. In another report, a 36-year-old woman with headache, paresis, positive neurological examination for ataxia of the upper limb, and decreased sensitivity to touch, temperature, and vibration on the left side, while receiving treatment with intrathecal nicardipine, she obtained an improvement of the arterial vasospasm evidenced on angiography [77].

In addition to BCCs, headache is controlled by analgesics, such as aspirin, although there is still no strong evidence of the clinical efficacy of this use [17]. In another case report, intravenous milrinone was described with therapeutic success for RCVS associated with eclampsia and refractory to nimodipine. Efficacy is believed to be due to its anti-inflammatory effect and the vasodilatory effect of the phosphodiesterase type 3 inhibitor [78]. In addition, the use of IA vasodilating agents or angioplasty for the most severe cases has also been described. However, experience with these modalities is limited, and the benefit is unclear [5]. Although previously considered a potential treatment, corticosteroid use has had poor results in studies [1, 9, 13, 14, 28]. In addition, there is evidence of worsening RCVS soon after administering corticosteroids [13, 28, 39].

Therefore, it is worth noting that no guidelines or data from randomized studies are available in the literature to guide the management of RCVS effectively [11, 20].

### Complications

Reversible cerebral vasoconstriction syndrome has several complications, which include PRES, epileptic seizures, ischemic stroke, SAH, and intraparenchymal hemorrhage (IPH) [3, 13, 14, 22, 36, 67, 79]. Such complications are present in about 25–33% of patients with RCVS [3, 13, 14]. One study observed an incidence of PRES in 38% of patients, epileptic seizures in 17%, stroke in 39%, SAH in 34%, and IPH in 20%. Furthermore, it demonstrated that 20% of patients had persistent neurological deficits [5]. Furthermore, cerebral edema was present in 38% of patients, death due to stroke in 2% [2], and signs of cerebral infarction and cerebral hemorrhage in approximately 10–20% of patients [80].

Posterior reversible encephalopathy syndrome, one of the most reported complications in the articles read in this review, has an incidence in RCVS ranging from 8% to 38% [5, 6, 10, 20, 28, 33, 43, 66]. It is associated with increased blood flow, leading to endothelial dysfunction, cerebral edema, and hypoperfusion [20].

Ischemic and hemorrhagic complications have also been reported in many studies in the scientific literature, leading to persistent neurological deficits and death in more severe cases [5, 10, 20, 28, 30, 43]. Typically, these bleeding events occur during the first week and ischemic complications later, around the second week [1]. Convex SAH, which can manifest unilaterally or bilaterally, was seen in about 33% of RCVS cases [10], reaching 43% in some studies [5, 43].

Stroke is also a complication associated with RCVS and usually occurs up to 3 weeks after the syndrome [67, 79]. It has an incidence of 4% in patients with RCVS treated at health services, such as clinics and the emergency sector, and 39% in inpatient hospital settings, which reveals that RCVS is more severe in this environment [37]. In addition, patients with stroke and RCVS usually present with more severe manifestations, permanent neurological deficits, and even death [37]. Therefore, this complication is an essential indicator of a worse prognosis of RCVS [40].

Cognitive impairment, which may result from stroke, has been little described in the literature. In a case report, a 36-year-old female patient, after being diagnosed with RCVS, presented deficits in autobiographical memory, cognitive flexibility, verbal and non-verbal learning, and information processing. The involvement was verified bilaterally in the frontal and temporal lobes and in the same region of the vasoconstriction observed in the MRI [7].

### Prognosis

The prognosis of RCVS is uncertain, but most patients have self-limiting manifestations [1, 11, 28] and a benign prognosis [40, 44, 54, 81], with 90–95% of patients recovering within weeks. Headache is reported to resolve within 3 weeks [7, 43], and angiographic findings within 12 weeks [7, 9, 12, 43, 64]. Less than 10% of patients are generally left with permanent and severe deficits, and deaths occur in 1% [5, 9, 68, 82] to 5% [14].

In the literature, there are still reports of recurrence of RCVS [17, 30, 32, 62, 83], with associated factors like the history of migraine [25] and physical exercise as a triggering factor for thunderclap headache in the first episode of RCVS [32]. Therefore, for a good prognosis and a lower chance of recurrence, avoiding the use of vasoactive substances that interfere with the clinical management of RCVS, such as adrenergic and serotonergic drugs, may be effective [84]. However, recurrence cases generally have a favorable outcome without serious complications [30].

However, some patients remain with a chronic daily headache that is difficult to treat, since many medications to relieve these symptoms are triggering factors for RCVS [8]. Furthermore, infarction, hemorrhage, or neurological deficits denote a worse prognosis [5, 7, 14]. Notably, there are also descriptions of patients in the postpartum state with worse prognoses, with death in 20% and residual deficits in 30% [68]. Remains of neurological deficits are also part of a poor prognosis, being more common in patients who had RCVS after trauma or surgical procedure [85] and who had deficiencies in focal cortical areas, such as aphasia, apraxia, and neglect during the syndrome. Thus, patients with risk factors for a worse prognosis must be better observed [17].

## Conclusions

Due to the facts mentioned above, the main precipitating factors for RCVS are the postpartum state, nasal decongestants, and drugs with vasoactive properties. This understanding is fundamental to guide the treatment, since, initially, aggravating factors for the patient should be discontinued. In addition, although nimodipine is the drug of choice in most studies, in some cases, other drugs, such as verapamil and nicardipine, have better efficacy. The patient diagnosed with RCVS, especially combining the clinic with imaging tests, such as angiography, must undergo a thorough medical follow-up to avoid the appearance or, at least, the worsening of already known complications resulting from the syndrome.

## Data Availability

Not applicable.

## Abbreviations

CCBs: Calcium channel blockers
COVID-19: Corona virus disease 2019
CSF: Cerebrospinal fluid
CT: Cranial tomography
IA: Intra-arterial
IPH: Intraparenchymal hemorrhage
MRI: Magnetic resonance imaging
PACNS: Primary angiitis of the central nervous system
PRES: Posterior reversible encephalopathy syndrome
RCVS: Reversible cerebral vasoconstriction syndrome
SAH: Subarachnoid hemorrhage
